# Synthesis and Characterization of the First Tin Fluoride Borate Sn_3_[B_3_O_7_]F with Second Harmonic Generation Response

**DOI:** 10.1002/chem.201803478

**Published:** 2018-09-05

**Authors:** Sandra Schönegger, Stephan G. Jantz, Andreas Saxer, Lkhamsuren Bayarjargal, Björn Winkler, Florian Pielnhofer, Henning A. Höppe, Hubert Huppertz

**Affiliations:** ^1^ Institut für Allgemeine, Anorganische und Theoretische Chemie Leopold-Franzens-Universität Innsbruck Innrain 80–82 6020 Innsbruck Austria; ^2^ Institut für Konstruktion und Materialwissenschaften Leopold-Franzens-Universität Innsbruck Technikerstraße 13 6020 Innsbruck Austria; ^3^ Institut für Geowissenschaften Universität Frankfurt Altenhöferallee 1 60438 Frankfurt/Main Germany; ^4^ Institut für Anorganische Chemie Universität Regensburg Universitätsstraße 31 93053 Regensburg Germany; ^5^ Institut für Physik Universität Augsburg Universitätstr. 1 86159 Augsburg Germany

**Keywords:** borate, hydrothermal synthesis, SHG measurements, structure elucidation, thermoanalytical measurements, tin fluoride borate

## Abstract

The new non‐centrosymmetric tin fluoride borate Sn_3_[B_3_O_7_]F was synthesized hydrothermally, and was characterized by single‐crystal and powder X‐ray diffraction, vibrational spectroscopy, DFT calculations, second harmonic generation (SHG) measurements, thermogravimetry, and differential scanning calorimetry. Its SHG response is about 12 times that of quartz. The compound crystallizes in the non‐centrosymmetric orthorhombic space group *Pna*2_1_ with lattice parameters *a=*922.4(2), *b=*769.8(4), and *c=*1221.9(6) pm (*Z=*4). Characteristic for the structure are isolated B_3_O_7_ moieties, consisting of two corner‐sharing BO_3_ units and one BO_4_ tetrahedron. These occupy half of the octahedral voids of a slightly distorted hexagonal closest packing of Sn^2+^ atoms, with [SnF]^+^ units in the other half of the octahedral voids. Sn_3_[B_3_O_7_]F is transparent over a wide spectral range with a UV cut‐off edge at about 263 nm.

## Introduction

At the beginning of the 1960s, shortly after the demonstration of the first laser, Franken et al.^1^ observed for the first time second harmonic generation (SHG) when illuminating a quartz crystal with a ruby laser. This has triggered the on‐going search for non‐linear optical (NLO) materials suitable for a very broad range of applications. However, the challenge remains to obtain new NLO materials having a combination of desirable properties, such as large SHG coefficients, moderate birefringence, transparency over a broad spectral range for phase matching, a high damage threshold, good chemical stability, and facile crystal growth.^2^, ^3^, ^4^

Borates are of great interest as NLO materials as the boron atom in borates possesses the ability to coordinate either three or four oxygen atoms to form trigonal planar [BO_3_]^3−^ or tetrahedral [BO_4_]^5−^ units. These building units within a chiral structure adopt a coplanar configuration to promote SHG and birefringence. A variety of NLO borates are already known, including β‐BaB_2_O_4_ (BBO),^5^ LiB_3_O_5_ (LBO),^6^ CsB_3_O_5_ (CBO),^7^ CsLiB_6_O_10_ (CLBO),^8^, ^9^ and YCa_4_O(BO_3_)_3_ (YCOB).^10^ A breakthrough in the area of deep‐UV NLO crystals was achieved with the discovery of KBe_2_BO_3_F_2_ (KBBF),^11^ because it is the only material that can produce coherent light by direct SHG at wavelengths below 200 nm. The distinctive crystal structure of KBBF results in excellent NLO properties, such as moderate SHG coefficients, a wide viewing window, and a good birefringence. Further fluoride borates include Pb_3_B_6_O_11_F_2_,^12^ Ba_3_B_6_O_11_F_2_,^13^ Cd_5_(BO_3_)_3_F,^14^ and the fluorooxoborates BiB_2_O_4_F,^15^ NaB_4_O_6_F,^16^ SrB_5_O_7_F_3_,^17^ CsB_4_O_6_F^18^ have been reported, which also show SHG. All these compounds were synthesized using high‐temperature synthesis methods, with Pb_3_B_6_O_11_F_2_ being an exception as it was produced under hydrothermal conditions at 240 °C. Furthermore, the first fluorooxoborate Sn[B_2_O_3_F_2_] was also obtained recently.^19^ Compared to our fluoride borate, Sn[B_2_O_3_F_2_] represents a fluorooxoborate.

Our group has already succeeded in synthesizing new tin borates in the Sn‐B‐O‐H system like Sn_2_B_3_O_6_(OH)^20^ and SnB_8_O_11_(OH)_4_.^21^ Guided by the idea to synthesize the first tin fluoride borate under hydrothermal conditions in the system Sn‐B‐O‐F, we successfully obtained the novel compound Sn_3_[B_3_O_7_]F.

## Results and Discussion

### Crystal structure

**Figure 1:**
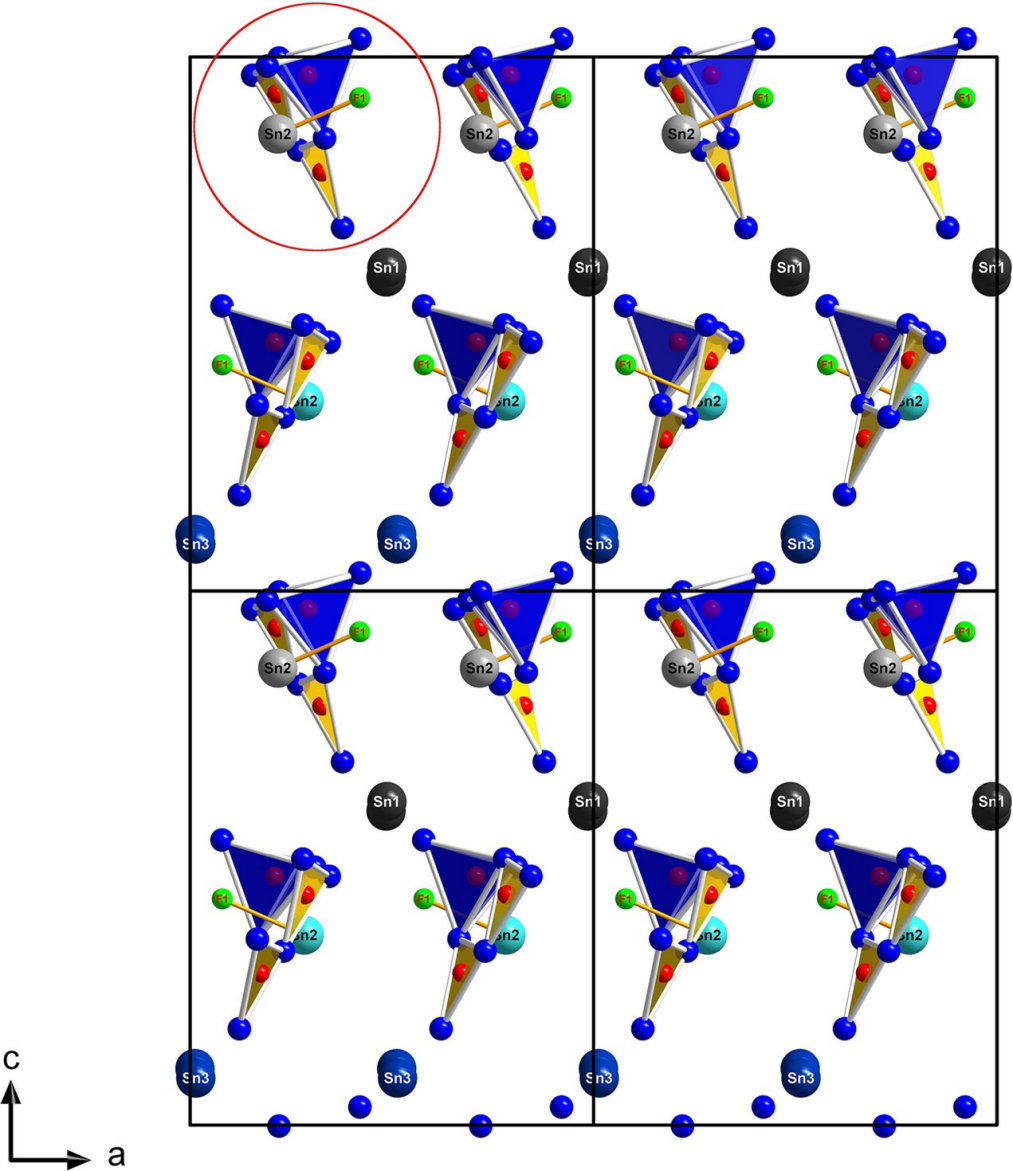
2 × 2 unit cell along the *b* axis in the crystal structure of Sn_3_[B_3_O_7_]F. The three‐membered ring ⟨□2Δ⟩ forming the fundamental building block (FBB) encircled in red. O^2−^: small blue spheres; F^−^: green spheres, B^3+^: small red spheres, and the three Sn^2+^ cations: dark blue, dark grey and light blue. BO_4_ groups are indicated with blue polyhedra and the BO_3_ groups with yellow triangles.

**Figure 2:**
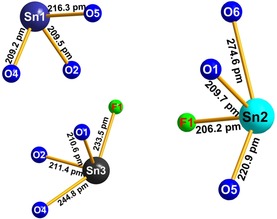
The three Sn^2+^ cations in Sn_3_[B_3_O_7_]F with the interatomic distances.

The crystal structure of Sn_3_[B_3_O_7_]F contains B_3_O_7_ groups forming three membered rings. The fundamental building block (FBB) consists of two corner‐sharing BO_3_ triangles and one BO_4_ tetrahedron, described as ⟨□2Δ⟩ 22 with the triangle and the square as pictograms for the trigonal‐planar [BO_3_]^3−^ and the tetrahedral [BO_4_]^5−^ group, respectively (see Figure 1, encircled in red). Interestingly, the tips of the BO_3_ groups are oriented once to the right (upper three membered rings) and once to the left (lower three membered rings). The Sn1 and Sn3 cations form a slightly distorted hexagonal closest packing with the three membered rings ⟨□2Δ⟩ in one half of the octahedral voids; the other half of the octahedral voids are filled with [SnF]^+^, as illustrated in Figure 2 and Figure S4 (Supporting Information). All these non‐centrosymmetric ⟨□2Δ⟩ rings are oriented in the same manner, resulting in a polar crystal structure along the *c* axis.

**Figure 3:**
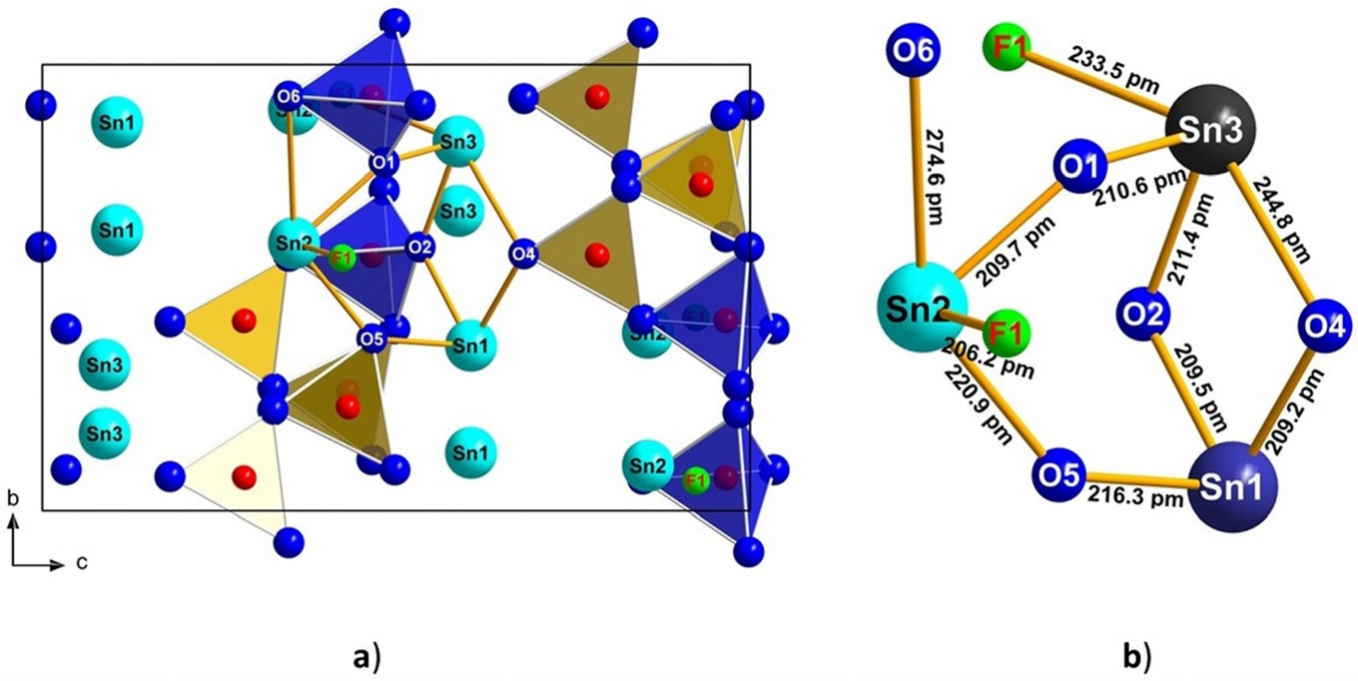
a) The unit cell of Sn_3_[B_3_O_7_]F along the *a* axis. In the centre of Figure 3 a, the coordination between the Sn^2+^ cations (light blue spheres), the O^2−^ atoms (dark blue spheres), and the F^−^ anions (yellow spheres) are presented; b) Enlarged representation of the coordination between the Sn, O, and F atoms, with the corresponding interatomic distances.

In the crystal structure of Sn_3_[B_3_O_7_]F, there are three unique boron atoms. The boron atom B2 is tetrahedrally coordinated by four oxygen atoms. The B−O distances in the tetrahedron range between 145.9(5)–148.8(5) pm, in good agreement with the average value of 147.6 pm.^22^, ^23^ The O‐B‐O bond angles in the [BO_4_]^5−^ group exhibit an average value of 109.5°, indistinguishable within error from the ideal tetrahedral angle of 109.47°. The atoms B1 and B3 are threefold coordinated by oxygen atoms forming BO_3_ groups. The values of the O‐B‐O bond angles range from 117.1(4) to 122.3(4)° (mean value=120.0°) and the B−O distances from 135.1(5) to 138.4(3) pm with an average value of 136.7 pm. This value agrees very well with the mean B−O distance of 137 pm for this coordination.^22^ Furthermore, there are three crystallographically unique Sn atoms, which possess different coordination environments (see Figure 2). The Sn1 cation coordinates three oxygen atoms, O2, O4, and O5 with Sn−O distances of 209.0(3)–216.3(3) pm (average value: 211.7 pm). The Sn2 and Sn3 cations coordinate three oxygen atoms and to the fluoride atom. Sn2 coordinates the oxygen atoms O1, O5, and O6 in the range of 209.7(3)–274.6(3) pm (average value: 235.1 pm), and to F1 with an interatomic distance of 206.2(3) pm corresponding to a Sn−F single bond. The Sn3 cation coordinates to the oxygen atoms O1, O2, and O4 with Sn‐O distances in the range of 210.7(3) to 244.8(3) pm (average value: 222.1 pm), and to F1 with an interatomic distance of 233.5(3) pm. From Figure 2 it is obvious that all Sn^2+^ cations exhibit a stereochemically active 5s^2^ lone electron pair directed into the open flank of each coordination environment; the O‐Sn‐X (X=O, F) angles of the closest three neighbours just below 90° (76.2(1)‐90.8(1)°) indicate the p character of the bonding interactions. Figure 3 a represents the structure of Sn_3_[B_3_O_7_]F along the *a* axis, where the connections between the Sn, O, and F atoms are shown. Figure 3 b shows the connectivity of the three tin atoms with the corresponding oxygen atoms and the unique fluoride atom in enlarged form with the relevant interatomic distances. Sn1 and Sn3 are interconnected via the two oxygen atoms O2 and O4, like in the compound Sn_2_B_3_O_6_(OH).^20^ The Sn−O distances are in the range of 209.2(3) to 244.8(3) pm.

The charge distribution of the atoms in the compound Sn_3_[B_3_O_7_]F was calculated according to the BLBS (bond‐length/bond‐strength, ∑*V*)^24^, ^25^, ^26^, ^27^, ^28^ and the CHARDI (charge distribution in solids) concept (∑*Q*). The results are listed in Table S3 given in the Supporting Information, and are consistent with the formal valence states of the cations and anions.

### Vibrational spectroscopy

**Figure 4:**
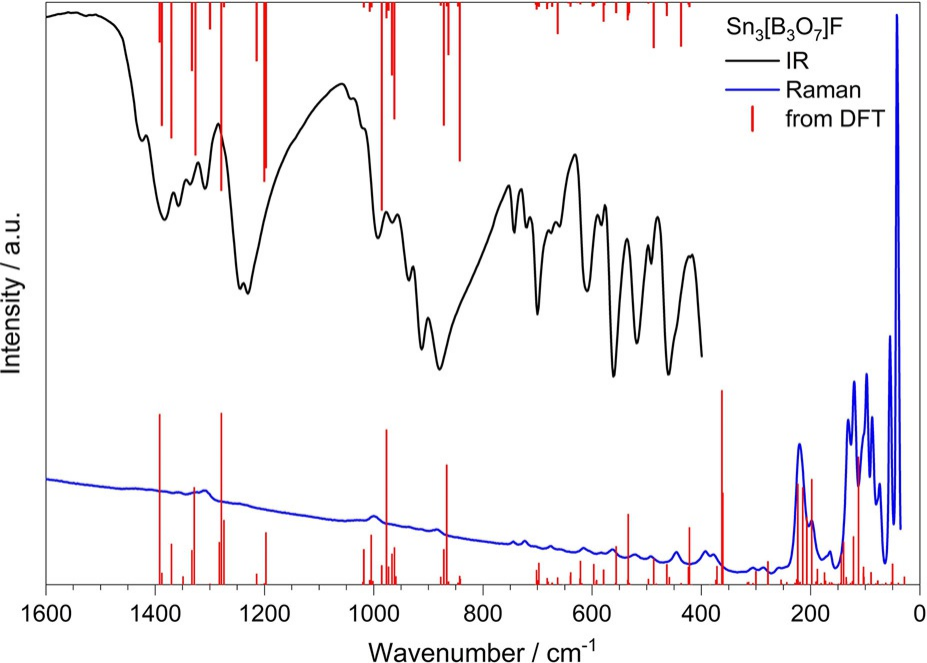
IR (black) and Raman (blue) spectra of Sn_3_[B_3_O_7_]F. The red bars denote band positions calculated by DFT.

Figure 4 shows the experimental IR and Raman spectra recorded from powder samples, in comparison to the results of density functional theory‐based calculations. An extended IR spectrum (4000 to 400 cm^−1^) is shown in Figure S1 in the Supporting Information. Above 1600 cm^−1^ no bands were recorded, which is a very strong indication for the absence of hydroxyl groups. The existence of triangular BO_3_ and tetrahedral BO_4_ groups in the structure was confirmed. The strong bands between 1600 and 800 cm^−1^ are typical B−O stretching vibrations, whereby the bands of [BO_3_]^3−^ vibrations are expected between 1450 and 1150 cm^−1^,^29^, ^30^, ^31^ and the absorption peaks of the asymmetric and symmetric stretching modes of the BO_4_ groups are located in the range between 1050 and 800 cm^−1^.^30^, ^31^, ^32^ The bands between 800 and 400 cm^−1^ can be assigned to bending vibrations of BO_3_ and BO_4_ groups,^33^ as well as ring breathing modes. The experimental Raman spectrum suffers from very low intensities above 250 cm^−1^ and allows only the determination of the frequencies of lattice vibrations in the range 250 to 35 cm^−1^. According to group theory, the irreducible representations of Sn_3_[B_3_O_7_]F are 42A_1_+42A_2_+42B_1_+42B_2_. The three acoustic modes have the irreducible representations A_1_+B_1_+B_2_. All optical modes (Γ_Raman_=41A_1_+42A_2_+41B_1_+41B_2_) are Raman active. The infrared active modes have representations Γ_IR_=41A_1_+41B_1_+41B_2_ and all A_2_ modes are infrared inactive.

### UV/Vis‐NIR spectroscopy

**Figure 5:**
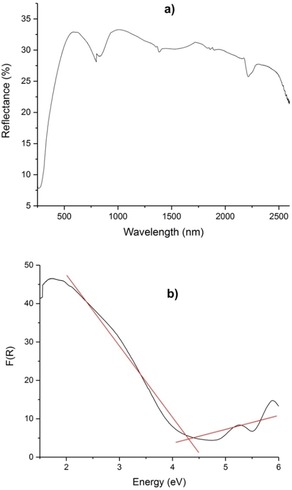
a) UV/Vis‐NIR diffuse‐reflectance spectrum of Sn_3_[B_3_O_7_]F; b) The UV/Vis‐NIR absorption spectrum calculated from the reflectance spectrum for Sn_3_[B_3_O_7_]F.

Figure 5 a illustrates the UV/Vis‐NIR diffuse‐reflectance spectrum of Sn_3_[B_3_O_7_]F. Apparently, the cut off edge is lower than 263 nm, potentially making Sn_3_[B_3_O_7_]F suitable for UV NLO applications. Based on the UV/Vis‐NIR diffuse‐reflectance spectrum, the absorption (*K*/*S*) data were calculated from the Kubelka–Munk function [Eq. (1)]:^34^, ^35^, ^36^

(1) $$F\left(R\right)=\;\frac{{\left(1\;-R\right)}^{2}}{2R}\;=\;\frac{K}{S}$$

where R is the reflectance, K the absorption, and S is the scattering. Furthermore, the experimental band gap of 4.30 eV is shown in Figure 5 b.

### Thermal behaviour

**Figure 6:**
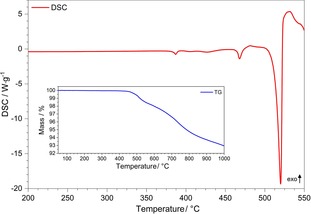
The simultaneous thermal analysis (STA) of Sn_3_[B_3_O_7_]F with the thermogravimetric curve (TG) in blue and the differential scanning calorimetry curve (DSC) in red.

To study the high‐temperature stability of Sn_3_[B_3_O_7_]F, thermosanalytical measurements were performed. Figure 6 illustrates the results from differential scanning calorimetry (DSC) and thermogravimetric analysis (TG), which were recorded from ambient temperature to 550 °C and 1000 °C, respectively. Sn_3_[B_3_O_7_]F is stable up to a temperature of 490 °C. There are three endothermic peaks in the investigated range. The first one, with an onset temperature of 380 °C, is probably due to the conversion of a small amount of Sn[B_2_O_3_F_2_] contaminant with concentrations below the detection limit of the powder XRD.^19^ The second peak, with an onset temperature of 465 °C, can be related to the melting point of the small amorphous boron oxide contamination which is also not detectable by powder XRD. The following thermal decomposition of Sn_3_[B_3_O_7_]F is an endothermic one‐step process with an onset temperature of 500 °C. The resulting decomposition product is currently still under investigation. The TG curve features at least two decomposition steps, which are poorly resolved. The onset temperature is 450 °C and the continuous mass loss is not finished at 1000 °C. Most probably this is due to the slow evaporation of B_2_O_3_, but this is still under investigation. In addition, thermoanalytical measurements were performed on a sample of Sn_3_[B_3_O_7_]F with Sn[B_2_O_3_]F_2_ as an impurity (see Figure S2 in Supporting Information), showing a slightly different thermal behaviour (due to the mixture): already at 400 °C, a large endothermic maximum could be found, which was accompanied by a mass loss of 5.4 %, matching quite well with the calculated mass loss for BF_3_ (calculated: 4.4 %). Furthermore, HT‐PXRD data were collected on this sample revealing the decomposition of Sn_3_[B_3_O_7_]F (containing Sn[B_2_O_3_F_2_] as an impurity) to SnO_2_ and a glassy, amorphous phase, which was indicated by the small halo at middle theta angles (see Figure S3). Attempts to isolate and crystallize an intermediate product of the thermal decomposition were not successful.

### Second harmonic generation measurements

The SHG measurements were performed on an ungraded powder of Sn_3_[B_3_O_7_]F employing the powder SHG method,^37^ which is commonly used in the first step to estimate the nonlinear optical properties of new SHG materials. The SHG intensity of Sn_3_[B_3_O_7_]F was 5080(1066) counts and these second‐harmonic signal is found to be ≈12 times that of quartz and ≈0.4 times that of KH_2_PO_4_ (KDP). This strong SHG signal implies that the sample has large SHG coefficients or is phase matchable. The centrosymmetric reference sample Al_2_O_3_ shows a SHG intensity of 4(10) counts. Table 1 illustrates the intensities of the Sn_3_[B_3_O_7_]F sample and all three reference materials. The point group *mm*2 has five independent SHG coefficients (*d*_31_, *d*_32_, *d*_33_, *d*_15_, and *d*_24_).

**Table 1:** SHG intensities of the Sn_3_[B_3_O_7_]F sample compared with the three reference materials.

Samples	SHG intensity in counts
Al_2_O_3_	4(10)
Quartz	420(54)
Sn_3_[B_3_O_7_]F	5080(1066)
KDP (KH_2_PO_4_)	12953(1512)

### DFT calculation

Quantum chemical calculations in the framework of density functional theory (DFT) were performed to investigate the electronic structure of Sn_3_[B_3_O_7_]F, in order to interpret the experimentally determined vibrational spectra. The obtained vibrational frequencies are in very good agreement with the experimental values as shown in Figure 4 and discussed above. The lattice parameters obtained by full geometry optimisations are slightly overestimated as a consequence of the PBE exchange‐correlation functional employed here with *a=*954, *b=*772, and *c=*1222 pm. In this context, it should be mentioned that DFT‐PBE usually overestimates lattice parameters and underestimates optical band gaps.

From electronic band structure calculations, an indirect band gap of 3.8 eV, which underestimates the experimental optical band gap of 4.3 eV, is obtained. This is a common and well‐known effect of the standard functionals in DFT. Further, partial densities of states (PDOS) point out that the valence band mainly consists of Sn 5s, Sn 5p, and O 2p orbitals, whereas the conduction band is almost exclusively formed from Sn 5p contributions (see Figure S5).

**Figure 7:**
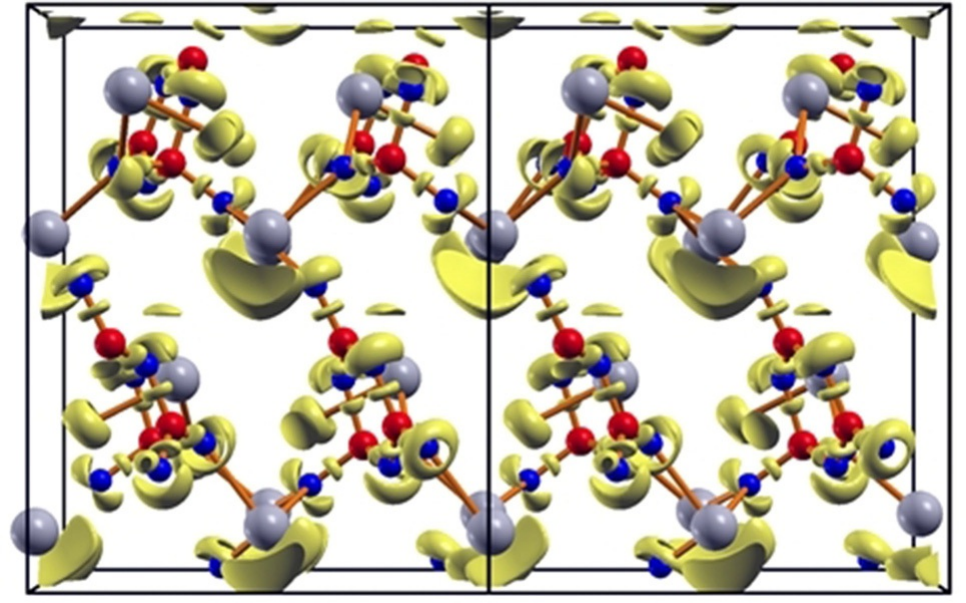
Electron localization function (ELF) (isosurface at 0.85) showing the attractors of the B−O bonds, O lone pairs, F lone pairs, and structure directing lone pairs of Sn^2+^. Colour code: Isosurface (yellow), boron (red), oxygen (blue), tin (grey); the fluorine atoms are not visible due to the overlapping ELF isosurface.

Real space bonding analysis through the electron localization function (ELF) was carried out to visualize covalent bonds and lone pairs of the title compound. High values of the ELF (0.85) appear in regions of the covalent B−O bonds and further highlight the stereochemically active lone pairs of the Sn^2+^ cations, as well as the lone pairs of the O and F atoms (Figure 7).

## Conclusion

Sn_3_[B_3_O_7_]F is the first compound in the system Sn‐B‐O‐F, which was synthesized by a straightforward, cost‐efficient hydrothermal synthesis. The structure contains isolated B_3_O_7_ rings. Between layers of these isolated three membered rings, the Sn1 and Sn3 cations are alternatingly ordered. The Sn2 cations and the F anions are located in a row with the B_3_O_7_ units.

## Experimental Section

### Synthesis

**Figure 8:**
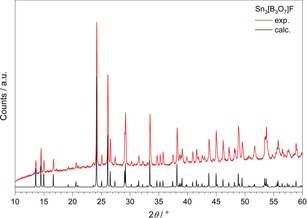
Experimental powder pattern (Cu_*Kα*_ radiation; *λ*=154.056 pm) (top), compared with the calculated powder pattern of Sn_3_[B_3_O_7_]F (bottom).

Sn_3_[B_3_O_7_]F was synthesized hydrothermally in a stainless‐steel autoclave (volume: 8 mL) with a Teflon insert. A mixture of SnO [107 mg, 0.8 mmol, Strem Chemicals, Inc. (≥ 99.8 %, Newburyport, USA)], SnF_2_ [78 mg, 0.5 mmol, p.a., Alfa Aesar (Karlsruhe, Germany)], and H_3_BO_3_ [119 mg, 1.9 mmol, Roth GmbH + Co. KG (≥ 99.8 %, Karlsruhe, Germany)] was heated up to a temperature of 510 K and kept there for 4 days. Afterwards, the reaction mixture was cooled down with a rate of two degrees per hour down to 330 K and finally quenched to room temperature. A light greyish powder was obtained and analysed by X‐ray powder diffraction. Furthermore, transparent, colourless platelets of Sn_3_[B_3_O_7_]F were isolated and measured by single‐crystal X‐ray diffraction. Via this route, the compound Sn_3_[B_3_O_7_]F could be synthesized with only a minor contamination by Sn[B_2_O_3_F_2_]. According to Figure 8, a phase pure product could be obtained via an alternate synthesis starting from Sn[B_2_O_3_F_2_],^19^ which decomposes around 400 °C yielding the title compound in the form of large single‐crystals. This is consistent with the results of HT‐XRD (Figure S3 in Supporting Information), which show that the reflections of the impurity phase Sn[B_2_O_3_]F_2_ vanish at 400 °C.

### Crystal structure analysis

The product Sn_3_[B_3_O_7_]F was identified by powder X‐ray diffraction data collected with a Seifert XRD T/T 3003 powder diffractometer in reflection geometry with Cu_*Kα*_ (*λ*=154.056 pm) radiation, equipped with a Meteor 1D linear detector. Figure 8 shows the experimental powder pattern of Sn_3_[B_3_O_7_]F at the top compared to the theoretical pattern derived from the single‐crystal data. For the single‐crystal structure analysis, a colourless platelet of Sn_3_[B_3_O_7_]F was isolated through polarization contrast microscopy and analysed via single‐crystal X‐ray diffraction. The intensity data were collected at 180 K with a Bruker D8 Quest diffractometer (Photon 100) equipped with an Incoatec Microfocus source generator (multi layered optics monochromatized Mo_*Kα*_ radiation, *λ*=71.073 pm). Multi‐scan absorption corrections were applied with the program SADABS‐2014/5.^38^

After structure solution and parameter refinement with anisotropic displacement parameters for all atoms using the SHELXS/l‐13 software suite,^39^, ^40^ the space group *Pna*2_1_ was found to be correct. All atoms were refined with anisotropic displacement parameters. All relevant details of the data collections and evaluations are shown in the Tables 2, Table 3, and Table 4. Tables S1 and S2 given in the Supporting Information show the positional parameters and the anisotropic displacement parameters, respectively. Further details of the crystal structure investigation(s) may be obtained from Fachinformationszentrum Karlsruhe, 76344 Eggenstein‐Leopoldshafen, Germany (fax: (+49)7247‐808‐666; e‐mail: crysdata@fiz‐karlsruhe.de, https://www.fiz‐Karlsruhe.de/en/leistungen/kristallographie/kristallstrukturdepot/order‐form‐request‐for‐deposited‐data.html) on quoting the deposition number CSD‐434719.

**Table 2:** Crystal data and structure refinement of Sn_3_[B_3_O_7_]F (standard deviations in parentheses).

empirical formula	Sn_3_B_3_O_7_F
molar mass [g mol^−1^]	519.5
crystal system	orthorhombic
space group	*Pna*2_1_ (no. 33)
single‐crystal diffractometer	Bruker D8 QUEST PHOTON 100
radiation; wavelength [pm]	Mo_*Kα*_; *λ*=71.073
*a* [pm]	922.38(4)
*b* [pm]	769.78(4)
*c* [pm]	1221.88(6)
*V* [nm^3^]	867(7)
formula units per cell, *Z*	4
calculated density [g cm^−3^]	3.977
crystal size [mm]	0.055×0.035×0.015
temperature [K]	183(2)
absorption coefficient [mm^−1^]	8.59
*F*(000) [e]	920
2*θ* range [deg]	6.26–70.01
range in *hkl*	−14 ≤ *h* ≤14 −12 ≤ *k* ≤12 −19 ≤ *l* ≤19
total no. of reflections	24 503
independent reflections/*R*_int_	3820/0.0364
reflections with *I*>2 *σ*(*I*)	24 503
data/refined parameters	3820/127
absorption correction	multi‐scan (Bruker sadabs 2014/5)
goodness‐of‐fit on *F*_i_^2^	1.072
final *R*1/*wR*2 [*I*>2 *σ*(*I*)]	0.0217/0.0399
*R*1/*wR*2 (all data)	0.0256/0.0407
Flack parameter	0.016(1)

**Table 3:** Selected interatomic distances [pm] in Sn_3_[B_3_O_7_]F (standard deviations in parentheses).

Sn1‐O2	209.5(3)	B1‐O3	135.1(5)
Sn1‐O4	209.2(3)	B1‐O5	137.4(5)
Sn1‐O5	216.3(3)	B1‐O7	138.2(5)
	Ø=211.7		Ø=136.7
Sn2‐O1	209.7(3)	B2‐O1^1^	147.2(5)
Sn2‐O5	221.0(3)	B2‐O2	145.9(5)
Sn2‐O6	274.6(3)	B2‐O3^5^	148.1(5)
Sn2‐F1	206.2(3)	B2‐O6^5^	148.8(6)
	Ø=227.7		Ø=147.5
Sn3‐O1	210.7(3)	B3‐O4^6^	136.0(6)
Sn3‐O2	211.4(3)	B3‐O6	135.8(5)
Sn3‐O4	244.8(3)	B3‐O7	138.4(5)
Sn3‐F1^1^	233.5(3)		Ø=136.7
	Ø=225.1		
		F1‐Sn2	206.2(3)
		F1‐Sn3^2^	233.5(3)
			Ø=219.9

1 1/2+*x*,3/2−*y*,+*z*;^2^ 1/2+*x*,3/2−*y*,+*z*;^3^−1/2+*x*,1/2−*y*,+*z*;^4^ 1/2−*x*,1/2+*y*,1/2+*z*;^5^ 1/2+*x*1/2−*y*,+*z*;^6^ 1/2−*x*,−1/2+*y*,−1/2+*z*.

**Table 4:** Bond angles [°] for Sn_3_[B_3_O_7_]F (standard deviations in parentheses).

O2‐Sn1‐O5	82.5(1)	O3‐B1‐O5	121.2(4)
O4‐Sn1‐O2	76.2(1)	O3‐B1‐O7	121.6(4)
O4‐Sn1‐O5	90.5(1)	O5‐B1‐O7	117.1(4)
	Ø=83.1		Ø=120.0
O1‐Sn2‐O5	90.8(1)	O1^1^‐B2‐O3^5^	109.7(3)
F1‐Sn2‐O1	83.7(1)	O1^1^‐B2‐O6^5^	108.0(3)
F1‐Sn2‐O5	84.1(1)	O2‐B2‐O1^1^	111.0(3)
	Ø=86.2	O2‐B2‐O3^5^	107.1(3)
		O2‐B2‐O6^5^	109.8(3)
O1‐Sn3‐O2	88.8(1)	O3‐B2‐O6^5^	111.2(3)
O1‐Sn3‐O4	80.3(1)		Ø=109.5
O1‐Sn3‐F1^1^	79.8(1)		
O2‐Sn3‐O4	68.5(1)	O4^6^‐B3‐O7	117.5(4)
O2‐Sn3‐F1^1^	83.0(1)	O6‐B3‐O4^6^	122.3(4)
F1^1^‐Sn3‐O4	145.3(1)	O6‐B3‐O7	120.2(4)
	Ø=91.0		Ø=120.0

1 1/2+*x*,3/2−*y*,+*z*;^2^ −1/2+*x*,3/2−*y*,+*z*;^3^ −1/2+*x*,1/2−*y*,+*z*;^4^ 1/2−*x*,1/2+*y*,1/2+*z*;^5^ 1/2+*x*,1/2−*y*,+*z*;^6^1/2−*x*,−1/2+*y*,−1/2+*z*.

### Vibrational spectroscopy

An FTIR‐ATR (Attenuated Total Reflection) characterization of the Sn_3_[B_3_O_7_]F powder sample was performed in the spectral range of 400–4000 cm^−1^ with a Bruker ALPHA Platinum‐ATR spectrometer (Bruker, Billerica, USA) equipped with a 2×2 mm diamond ATR‐crystal and a DTGS detector. 320 scans of the powder sample were acquired and afterwards corrected for atmospheric influences using the Opus 7.2 software.^41^

### Raman spectroscopy

Raman spectra were recorded on a Thermo Scientific DXR Raman‐Microscope in the range 1800‐60 cm^−1^ using a 532 nm laser operated with 10 mW power. The sample was illuminated for 3600 s (10–fold magnification, 50 μm pinhole aperture, high resolution grating (1800 lines mm^−1^), spectral resolution 1 cm^−1^).

### UV/Vis‐NIR spectroscopy

The UV/Vis diffuse reflection data were recorded at ambient temperature. A powder sample of BaSO_4_ has been used as standard (100 % reflectance). The spectrum of the Sn_3_[B_3_O_7_]F powder was recorded from 190 to 2600 nm using an Agilent Cary 5000 UV/Vis‐NIR spectrophotometer at ambient temperature.

### Thermoanalytical investigation

The TG curve (Figure 7) was recorded on a TA Instrument Q500 TGA in a 90 mL min^−1^ nitrogen flow using an Al_2_O_3_ crucible; the DSC measurement was undertaken on a TA Instrument DSC 2920 in a 50 mL min^−1^ nitrogen flow using a Netzsch standard Al pan with pierced lid. Both measurements were performed with heating rates of 5 °C min^−1^.

### High temperature XRD

The high temperature X‐ray diffraction patterns were obtained on an Empyrean powder diffractometer (Panalytical) in theta‐theta geometry, equipped with a Cu tube with 40 kV tube tension and 40 mA tube current, and a Pixcel 1D detector. Furthermore, a variable divergence slit was used with an irradiated length of 6 mm. The sample was applied to the platinum strip (thickness of the platinum strip: 1 mm) and measured in an Anton Paar HTK 16N high temperature strip‐heater chamber under air and in a temperature range from 300 K to 870 K.

### Second harmonic generation measurements

According to the non‐centrosymmetric orthorhombic space group *Pna*2_1_ (no. 33), the SHG effect of the Sn_3_B_3_O_7_F compound was tested. A detailed description of the experimental setup for the powder SHG measurement is given by Bayarjargal et al.^42^ The SHG measurement was performed on a Q‐switched Nd:YLF laser system (Falcon 217D, Quantronix) operating at 1054 nm and with a pulse width of 130 ns, which provided the fundamental wave. The sample was placed on an adhesive tape, which did not generate a SHG signal. The SHG signal was detected at 527 nm. From five different areas of the sample, SHG measurements were performed with 15 measurements at each position, and the intensities of these five different positions were averaged, to check the homogeneity of the sample. The measured intensities were collected by subtracting a background signal, which was collected between the pulses. As reference materials, quartz (α‐SiO_2_), Al_2_O_3_, and KDP powders were used.

### DFT calculations

Quantum chemical calculations were performed in the framework of density functional theory (DFT) using a linear combination of Gaussian‐type functions (LCGTF) scheme as implemented in CRYSTAL14.^43^, ^44^ The total energy calculations including full structural optimizations were performed with the GGA (PBE)^45^ xc‐functional. The convergence criterion considering the energy was set to 1×10 a.u. with a k‐mesh sampling of 6×6×6. All‐electron basis sets were taken from references 46, 47, 48 and the outermost coefficients of the contractions were optimised. The vibrational frequencies, including Raman intensities, were computed on the basis of the relaxed structures using the coupled‐perturbed Kohn–Sham (CPKS) mode.^49^, ^50^ Vibrational modes were visualized with the J‐ICE application.^51^ The electronic structure was additionally assessed by the full potential local orbital (FPLO) method as implemented in the FPLO code (version 14.00–45).^52^ Scalar‐relativistic PBE calculations were carried out on a k‐mesh of 4×4×4. Further, a direct space analysis of the charge density was carried out by calculating the electron localization function (ELF) with TOPOND^53^ interfaced to CRYSTAL14. 3D plots were visualized with XCrysDen.^54^

## Conflict of interest

The authors declare no conflict of interest.

## Supporting information

As a service to our authors and readers, this journal provides supporting information supplied by the authors. Such materials are peer reviewed and may be re‐organized for online delivery, but are not copy‐edited or typeset. Technical support issues arising from supporting information (other than missing files) should be addressed to the authors.

Supplementary
