# Supplementary material for: Synthesis and Characterization of the First Tin Fluoride Borate Sn3[B3O7]F with Second Harmonic Generation Response
Source: Chemistry. 2018 Sep 5;24(60):16036–43. doi: 10.1002/chem.201803478 (PMC6221176; doi:10.1002/chem.201803478)
Supplement: Supplementary file 1 — Supplementary [file CHEM-24-16036-s001.pdf]

CHEMISTRY
A European Journal

Supporting Information

Synthesis and Characterization of the First Tin Fluoride Borate $Sn_3[B_3O_7]F$ with Second Harmonic Generation Response

Sandra Schönegger,[a] Stephan G. Jantz,[e] Andreas Saxer,[b] Lkhamsuren Bayarjargal,[c] Björn Winkler,[c] Florian Pielnhofer,[d] Henning A. Höppe,*[e] and Hubert Huppertz*[a]

chem_201803478_sm_miscellaneous_information.pdf

Table S1

Fractional atomic coordinates and equivalent isotropic displacement parameters U_{eq} (Å^2) for $Sn_3B_3O_7F$ (space group $Pna2_1$ no. 33). U_{eq} is defined as one third of the trace of the orthogonalized U_{ij} tensor (standard deviations in parentheses). All atoms are positioned on the same Wyckoff position 4*e* (*x, y, z*).

Atom	*x*	*y*	*z*	U_{eq}
Sn1	0.48787(3)	0.37034(4)	0.60574(2)	0.00791(6)
Sn2	0.28257(3)	0.60071(3)	0.35570(3)	0.00886(6)
Sn3	0.48581(3)	0.82726(4)	0.58799(3)	0.00838(6)
B1	0.3088(3)	0.7808(4)	0.4830(2)	0.0074(5)
B2	0.5792(3)	0.5917(4)	0.5337(2)	0.0090(6)
B3	0.2796(3)	0.0916(4)	0.4980(3)	0.0088(5)
O1	0.3779(4)	0.5764(4)	0.6803(3)	0.0126(6)
O2	0.3449(4)	0.3855(4)	0.4657(3)	0.0106(6)
O3	0.1667(3)	0.9291(3)	0.3482(3)	0.0109(6)
O4	0.2315(4)	0.2258(4)	0.3258(3)	0.0151(7)
O5	0.2852(5)	0.2314(6)	0.4313(4)	0.0085(8)
O6	0.7084(5)	0.5731(6)	0.4658(4)	0.0066(8)
O7	0.1736(5)	0.0734(6)	0.2845(4)	0.0082(8)
F1	0.0798(3)	0.5670(4)	0.4236(2)	0.0153(6)

Table S2

Anisotropic displacement parameters (U_{ij} in Å^2) for $Sn_3B_3O_7F$ (space group *Pna*2_1 no. 33) with standard deviations in parentheses.

Atom	U_{11}	U_{22}	U_{33}	U_{23}	U_{13}	U_{12}
Sn1	0.01026(1)	0.00605(1)	0.00743(1)	0.00085(1)	-0.00099(1)	-0.00011(9)
Sn2	0.01219(1)	0.00785(1)	0.00652(1)	0.00058(1)	0.00167(1)	0.00038(1)
Sn3	0.00904(1)	0.00675(1)	0.00935(1)	-0.00203(9)	-0.00226(1)	0.00066(9)
B1	0.0063(1)	0.0049(1)	0.0110(1)	0.0003(1)	-0.0012(1)	-0.0001(1)
B2	0.0059(1)	0.0074(1)	0.0139(1)	-0.0004(1)	0.0031(1)	0.0004(1)
B3	0.0112(1)	0.0053(1)	0.0097(1)	0.0010(1)	-0.0022(1)	-0.0025(1)
O1	0.0204(2)	0.0103(1)	0.0072(1)	-0.0003(1)	0.0048(1)	0.0038(1)
O2	0.0139(2)	0.0064(1)	0.0116(1)	-0.0009(1)	-0.0033(1)	-0.0024(1)
O3	0.0152(1)	0.0073(1)	0.0102(1)	0.0020(1)	-0.0058(1)	-0.0016(1)
O4	0.0252(2)	0.0081(1)	0.0118(2)	0.0021(1)	-0.0094(1)	-0.0062(1)
O5	0.009(2)	0.007(2)	0.0098(2)	0.00120(2)	-0.0009(2)	-0.0016(2)
O6	0.0068(2)	0.0045(2)	0.0085(2)	-0.0004(1)	-0.0019(2)	0.0006(1)
O7	0.009(2)	0.007(2)	0.0088(2)	0.0006(2)	-0.0013(2)	0.0005(2)
F1	0.0085(1)	0.0170(1)	0.0204(1)	-0.0043(1)	0.0021(1)	-0.0012(1)

Table S3

Charge distribution in $Sn_3B_3O_7F$ (space group *Pna*2_1 no. 33) calculated with the bond-length/bond-strength concept (ΣV) [38, 39] and the CHARDI concept (ΣQ) on the left side.

	**Sn1**	**Sn2**	**Sn3**	**B1**	**B2**	**B3**	**O1**
**ΣV**	+2.10	+2.10	+2.04	+3.00	+3.03	+3.02	-2.21
**ΣQ**	+1.95	+1.80	+1.82	+3.13	+3.06	+3.24	-2.16
	**O2**	**O3**	**O4**	**O5**	**O6**	**O7**	**F1**
**ΣV**	-2.23	-1.79	-2.07	-2.15	-1.76	-1.93	-1.19
**ΣQ**	-2.13	-1.81	-1.92	-2.11	-1.78	-1.87	-0.82

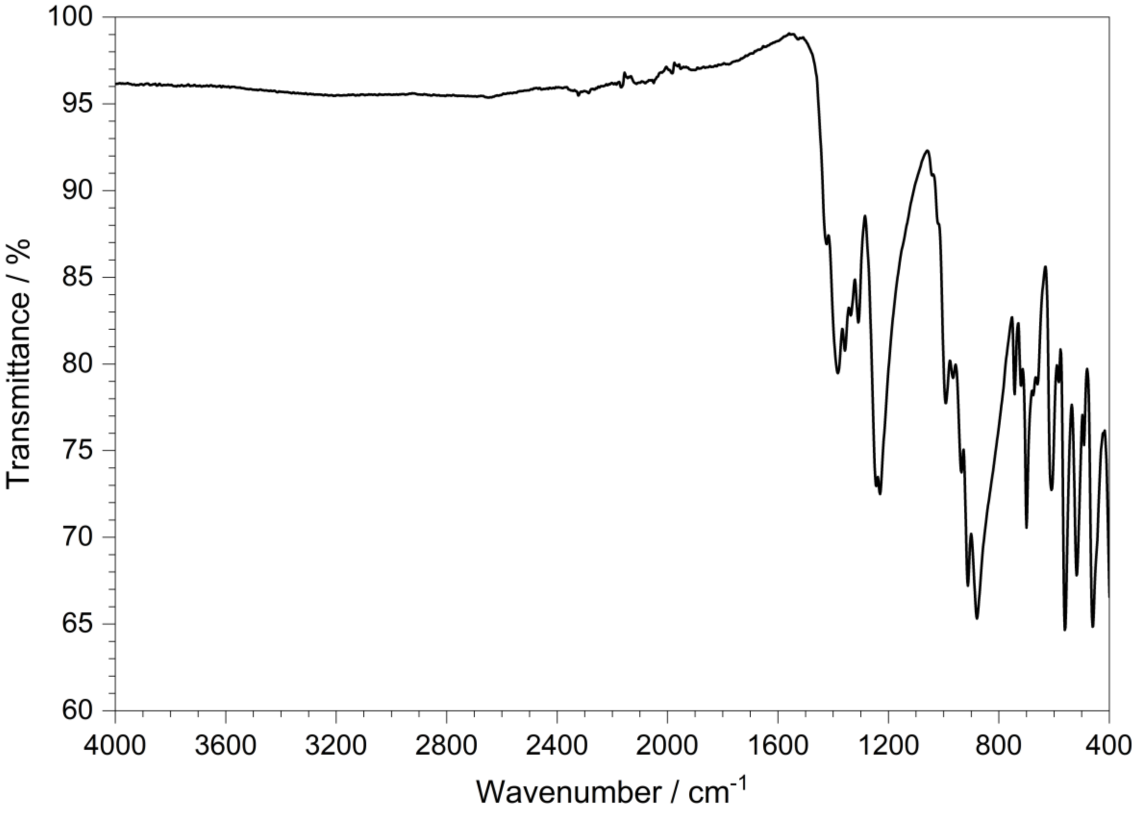

Fig. S1

Full range IR spectrum of $Sn_3[B_3O_7]F$.

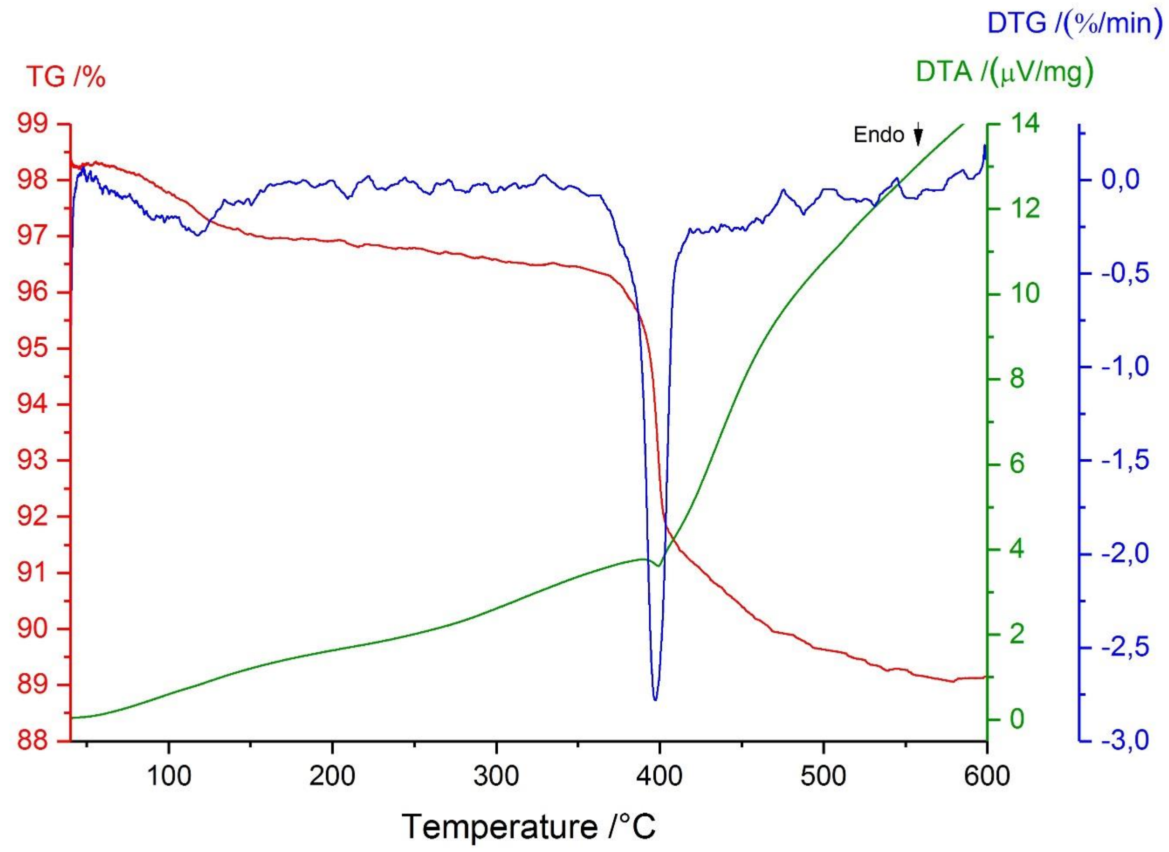

Fig. S2

The simultaneous thermal analysis (STA) of $Sn_3[B_3O_7]F$ with $Sn[B_2O_3]F_2$ as an impurity, with the thermogravimetric curve (TG) in red, the differential thermogravimetric curve (DTG) in blue, and the differential thermal analysis curve (DTA) in green.

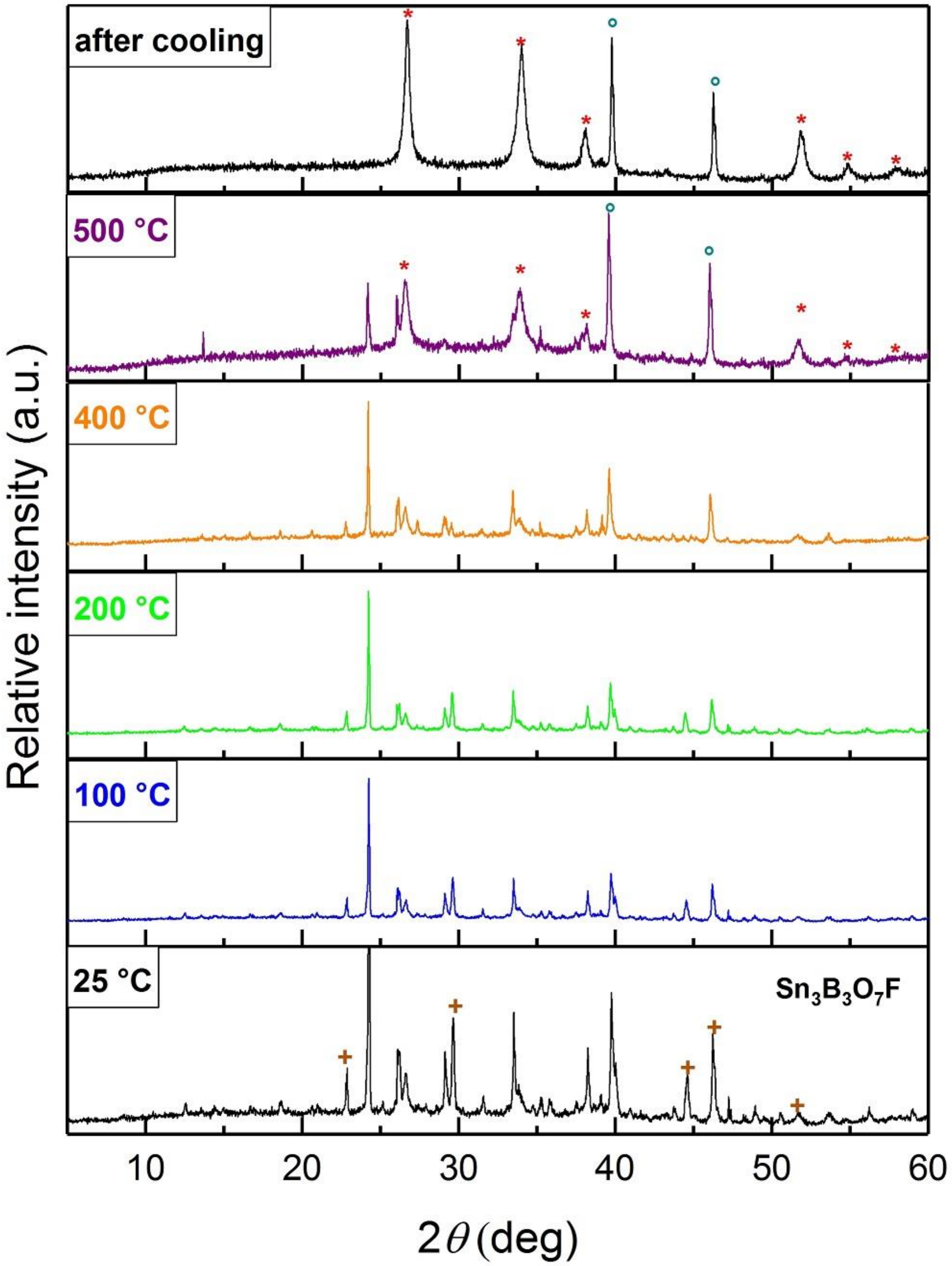

Fig. S3

The XRD patterns (Cu-K_α radiation, λ = 1.54178 Å) of $Sn_3[B_3O_7]F$ and its thermal decomposition at 25, 100, 200, 400, 500 °C, and after cooling. The reflections marked with a red asterisk in the patterns at 500 °C and after cooling stem from SnO_2; the dark green circle represents a reflection of the platinum heating strip, and the brown crosses represent the reflections of the side phase $Sn[B_2O_3]F_2$.

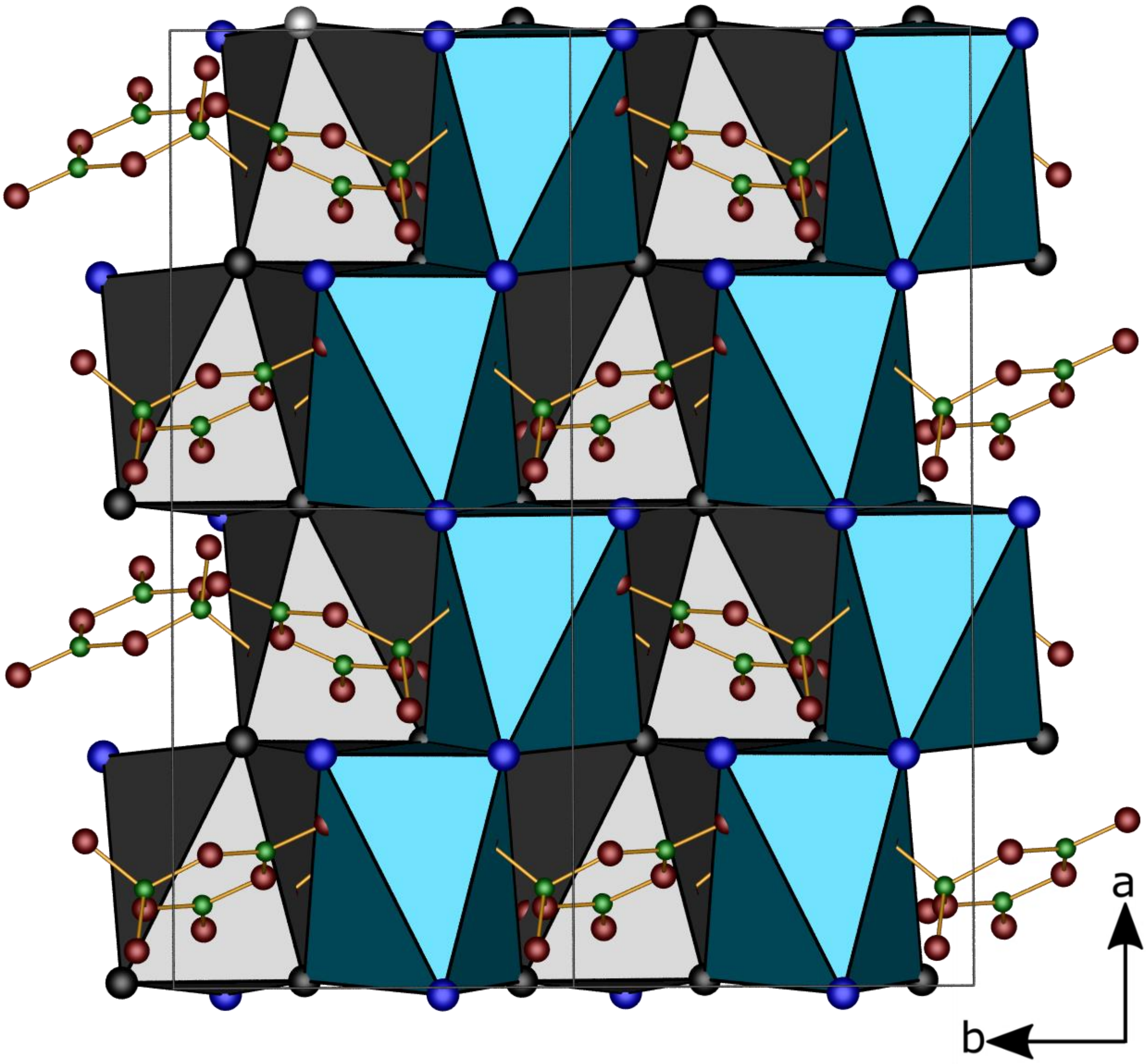

Fig. S4

Crystal structure of $Sn_3[B_3O_7]F$ along [001]; the octahedrons around the Sn3F1 dumbbells form edge-linked chains in the [100] direction (turquoise: O-layer; grey: O'-layer); Sn^{2+}: blue (A-layer) and black (B-layer), B^{3+}: green, and O^{2-}: red.

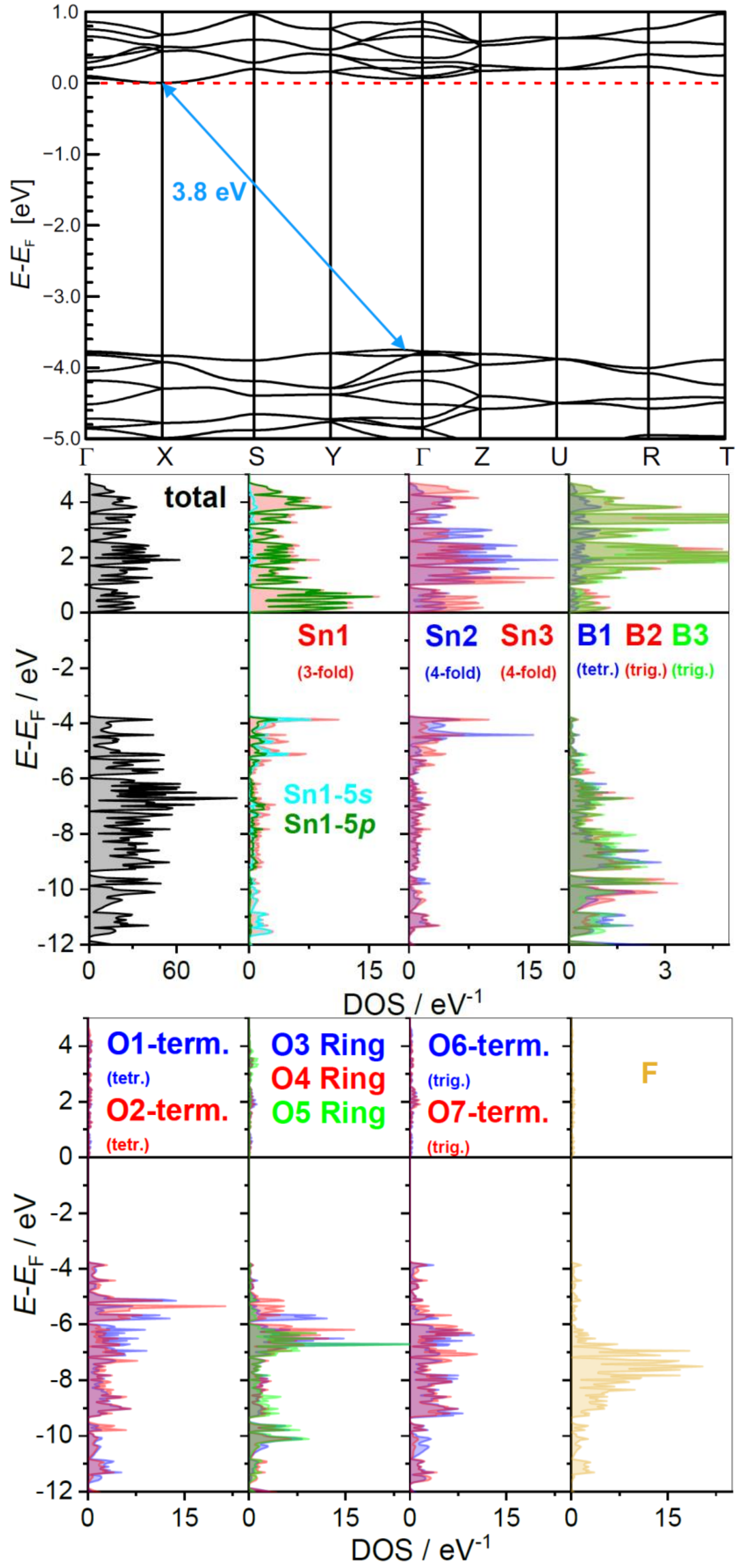

Fig. S5

Band structure calculations of $Sn_3[B_3O_7]F$; shown are the band structure comprising a calculated indirect band-gap as well as DOS and respective PDOS of all atoms.
